# Effect of Thermal Treatment on the Physicochemical, Ultrastructural, and Antioxidant Characteristics of *Euryale ferox* Seeds and Flour

**DOI:** 10.3390/foods11162404

**Published:** 2022-08-10

**Authors:** Qin Li, Hong-Tao Li, Yi-Peng Bai, Ke-Rui Zhu, Ping-Hsiu Huang

**Affiliations:** 1 School of Food Science and Technology, Jiangsu Food and Pharmaceutical Science College, 4 Meicheng Road, Huai’an 223003, China; 2 State Key Laboratory of Food Science and Technology, Jiangnan University, Wuxi 214122, China; 3 Number Times Technology (Huai’an) Co., Ltd., Huai’an 223113, China; 4 RuiDe Intelligent Technology (Huai’an) Co., Ltd., Huai’an 223113, China

**Keywords:** *Euryale ferox* seed, coarse grain, roasted, microwave, physicochemical, gelatinized, bioactive components, antioxidant

## Abstract

*Euryale ferox* seeds (EFS) were less gelatinized, preventing the release of nutrients and functional compounds, resulting in limited applications in meals and the food industry. Nutraceutical importance of EFS includes starch, protein, lipids, 20 amino acids, minerals, and vitamins (C, E, and beta carotene). This study aimed to evaluate the effect of three different thermal treatments on EFS’s physicochemical and nutritional properties and expected to improve its applicability. The results showed that the bulk density, thousand-grain weight, and hardness of thermal treated EFS were significantly decreased (*p* < 0.05), whereas the maximum decrease was observed in the industrial infrared heating-assisted fluidized bed (IHFH) treatment. Meanwhile, there were more crevices, fissures, and heightened porous structures in EFS between the pericarp and episperm and the endosperm after heat treatment, which facilitated grinding and water absorption. Notably, EFS’s water and oil absorption capacities increased significantly (*p* < 0.05) with microwave and IHFH treatments. EFS ground’s solubility into powder was increased significantly with thermal treatment (*p* < 0.05). Furthermore, the functional properties of TPC, TFC, DPPH radical scavenging activity, and reducing power were significantly increased (*p* < 0.05). In general, the changes in the physicochemical properties of EFS and increased bioactivity were caused by microwave and IHFH treatments. Hence, it might improve the food value of EFS while providing valuable information to researchers and food manufacturers.

## 1. Introduction

*Euryale ferox* Salisb., a large aquatic plant, is the only species of the Euryale genera and Nymphaeaceae family, found in India, Korea, Japan, Southeast Asia, and China [1,2]. There are some economic-scale cultivation and export trading in Jiangsu, Shandong, Hunan, Hubei, and Anhui provinces [2]. According to the change in people’s consumption concept, natural food with health care functions is favored by people more and more. Meanwhile, its cultivation areas are increasing, with high economic benefits and broad prospects. The common name of *Euryale ferox* seeds (EFS) is “Qianshi” in Chinese as well as cock’s head [3], which was originally published in the classic ”Shennong Herbal Medicine” and consumed medicinally or as food. It has high nutritional value and contains starch, protein, lipids, 20 amino acids (six essential amino acids, including leucine, isoleucine, lysine, threonine, valine, phenylalanine, and two other essential amino acids needed especially by children, histidine and arginine), minerals (Na, Mg, Ca, Se, P, Zn, Fe, Mn, Co, and Sn), vitamins (vitamins C and E and high amounts of beta carotene), and more active ingredients with health functions [4]. Notably, its starch granules are strongly bound and closely arranged. In China, it is widely served as food directly in soups or rice congee. In contrast, it is ground into powder for traditional foods such as steamed buns, cookies, and moon cakes, with even applications in fermented foods such as yogurt, vinegar, and functional beverages [2]. In addition, owing to the active ingredients mentioned above for health functions, it is primarily a traditional Chinese medicine with nourishing ingredients that have been used to treat diseases such as kidney problems, chronic diarrhea, excessive leucorrhea, and low spleen function [5]. Previous studies have shown that the seeds contain significant antioxidants and indicate closely related anti-inflammatory molecular mechanisms and anti-cancer machinery while possessing anti-fatigue, anti-depressant, and anti-diabetic activities [2,6,7,8,9]. Baek et al. [10] demonstrated that EFS contains tannins, tocopherol polymers, fucosterol, resorcinol, pyrogallol, cyclic dipeptides, glucosylsterols, cerebrosides, and polyphenols. However, plant polyphenols, which have a high capacity to trap reactive oxygen species and other free radicals, can reduce or prevent the production of oxidative reactions in tissues. They have been shown as possessing antioxidant, antibacterial, and antiviral activities. 

In developed countries, the trend in the food industry is for coarse grains to be processed into staple foods. In China, only about 30% of coarse grains appear on the table as staple foods, whereas the rest are used as raw materials for feed and extracted products. The primary reason is that the taste of coarse grain products is poor; thus, consumers’ acceptance is low. In addition, the dense structure and hard texture of EFS are not easy to crush. They are hard to gelatinize during heating, which affects the release of nutrients and functional compounds, which lead to the limitation of the application of EFS in the daily diet and food industry. In general, baking provides a more appealing aroma than other cooking methods. It can reduce the anti-nutritional factors of grains and legumes and increase the palatability of mixed grains with attractive sensory properties. The structural changes experienced by starch during thermal processing and its interaction with other macronutrients such as lipids and proteins are the main determinants of the flavor and texture of processed or cooked foods [11]. Moreover, it affects the digestibility of starch in processed foods. The microwave has some additional advantages over traditional methods, with minimal heating or cooking at high temperatures for a shorter period (microwave roasting) and then removing anti-nutritional factors, resulting in lower nutritional damage and higher quality final products [12]. It was found that the 30% moisture content of navy beans or chickpeas roasted at 160 °C increased the oil retention capacity of the flour by nearly 40% [13]. Rockembach et al. [14] showed that the primary benefits of the microwave parboiling process were increased gelatinization of 83% to 95%, and reduced browning of rice. A study that used microwave (60 s power 450 W) heat treatment of linseed (*Linum usitatissium* L.) grains showed no change in the amino acid distribution and content [15]. The economical, fast, and easy application of microwave roasting has further enhanced the gamut of roasted food products available worldwide. 

However, so far, to our knowledge, the detailed effects of heat treatment on EFS have not been evaluated. This study investigated the physicochemical, bioactive profile changes, and microstructure characteristics of EFS and roasted EFS flour with three different heat treatment processes. It aimed to improve the food value of EFS while providing valuable information to researchers and food manufacturers regarding its functional properties. 

## 2. Materials and Methods

### 2.1. Materials

The EFS was harvested in August 2021 and purchased from the local market (Huai’an, Jiangsu, China). Folin-Ciocalteu phenol reagent, sodium carbonate, 2,2-diphenyl-1-picrylhydrazyl (DPPH), Trolox, pyrogallol, rutin, chlorogenic acid, catechin, epicatechin, gallic acid, and vitamin C were purchased from Sigma (St. Louis, MO, USA). Methanol was of HPLC grade, while other reagents were of analytical grade. 

### 2.2. Thermal Treatment of EFS

The method was carried out according to Bai et al. [16] with minor modifications. As the possible effect of the difference in moisture content has been considered, the EFS was prepared to a moisture content of 14% and then treated by each of the three methods described below. 

Baking: The 200 g of EFS were weighed and spread on a 520 × 320 mm baking tray with a baking oven (SCC102, Rational Co., Landsberg am Lech, Germany) at 150 °C for 20 min.

Microwave treatment: The 50 g of EFS was placed in a glass dish (150 mm) and processed for 90 s at 900 W using a microwave oven (2450 MHz, 600–900 W, Midea, Guangdong Midea Kitchen Appliance Manufacturing Co., Ltd., China).

Industrial infrared heating-assisted fluidized bed (IHFH): according to the equipment supplier’s operating protocol, 5 kg of EFS per batch was poured into the IHFH (GW-100, Huai’an, China) at 150 °C for 20 min with a superficial airflow velocity of 6.0 m/s at optimum drying conditions.

The final moisture content of kernels treated with baking, microwave, and IHFH was 5.71 ± 0.20%, 5.24 ± 0.14%, and 7.99 ± 0.07%, respectively. All the kernels treated with the three methods were immediately subjected to a drying oven with a temperature of 35 °C to eliminate condensation of the residual steam and stabilize the grains. Finally, raw and thermal-treated EFS were ground through a high-speed grinding powder machine (FM100, Tianjin Tester Instrument Co. Ltd., Tianjin, China) and passed through a 0.18 mm sieve to acquire roasted EFS flours, which were kept in a sealed plastic bag and stored at room temperature (25 °C) until used.

### 2.3. Observation of Surface Morphology

Each sample of approximately 20 kernels was placed on a black plastic plate and then placed in a small PhotoStudio (Sutefoto, Shenzhen Sute Photographic Equipment Co., Ltd., Shenzhen, China; 400 × 400 × 400 mm) and recorded with a high-performance digital SLR camera (EOS 70D, Canon Corporation, Tokyo, Japan).

### 2.4. Measurement of Physicochemical Characteristics

The method was performed with modifications according to Bai et al. [16]. The EFS was filled into a 500 mL graduated measuring cylinder by tapping 30 times gently, and then the kernels were poured out to determine the weight (g), expressed as bulk density (g/L). The bulk density of the control sample was divided by the bulk density of the thermally treated EFS kernels to obtain the puffing index. The thousand-grain weight (g) means the measurement of 1000 raw or puffed EFS. The hardness was measured according to a previously described method [16]. A texture analyzer (TA-XT plus C, Stable Micro Systems Ltd., Surrey, UK) was used for the measurements. An EFS kernel was placed on the platform and compressed with a P/36R probe. The parameters set were test velocity, 1.00 mm/s post-test velocity, 10.00 mm/s; distance, 0.500 mm; and trigger force 5.0 g. The units of measure are Newtons.

### 2.5. Color Measurement

The color measurement of roasted EFS flour was carried out according to a previous method [17]. The variation of sample color was measured by a digital colorimeter (CR-400, Konica Minolta Optics, Inc., Tokyo, Japan), whereby the L* value is for lightness/darkness, the a* value for redness/greenness, the b* value for yellowness/blueness, and ΔE for the total color difference and the raw EHS flour has as the control group. The equation for color difference (ΔE) is as follows.

ΔE = [(L* − *L*_0_*)^2^ + (a* − *a*_0_*)^2^ + (b* − *b*_0_*)^2^] (1)

The subscript “_0_” indicates the color value of the raw EFS flour.

### 2.6. Scanning Electron Microscopy (SEM) Measurement

The microstructures of EFS and its seed coat were investigated using a scanning electron microscope (SU8100, Tokyo, Japan) according to a previous method [16]. Magnifications of 30×, 150×, 200×, and 1000× were applied for the cross-section observation, and a magnification of 1000× was adopted for the surface.

### 2.7. Low-Field Magnetic Resonance Imaging (MRI) Measurement

A total of 20 g of EFS grains were soaked in water at a ratio of 1:2 at 25 °C. One grain was taken out for measuring the T2-weighted images of EFS after soaking for 1 and 3 h by a low-field MRI system (MesoMR23-060V-I, Shanghai, China). Specifically, the sample was sealed using PTFE thread seal tape to prevent air and moisture and transferred to a 25 mm glass test tube. Then, the glass test tube was put in the center of the 0.5 ± 0.05 T permanent magnetic field coil. Next, the test pulse sequence was selected as multi-spin-echo (MSME). More specifically, the testing parameters were Echo Time (TE), 20.0 ms; Slice Width (SW), 5.0 mm; Repetition Time (TR), 500 ms; Average, 2; Field of View (FOV) = 50 × 50 mm, Test Temperature, 32 °C.

### 2.8. Water and Oil Absorption Capacities

The centrifuge tube was pre-weighed, and then 1.0 g of sample was added, next by 15 mL of distilled water or oil, with continuous stirring for 1 h at room temperature, followed by centrifugation (H2050R, Changsha, China) at 6000 rpm for 15 min. The centrifuged tube was tilted at 45 °C for 10 min and then poured out the free water or oil. Afterward, it was weighed for an increase in weight, representing the sample’s absorption of water or oil [18].

### 2.9. Swelling Power and Solubility

Swelling power and solubility of the prepared roasted flours were determined by the method of Dudu et al. [19] with slight modifications. First, we added 1.0 g of sample (dry base) to a pre-weighed centrifuge tube to be mixed uniformly with distilled water (15 mL). Next, it was heated in a water bath at 95 °C for 1 h (with 10 s vortexing every 10 min). When the reaction completed, it was cooled to room temperature in an ice bath and then centrifuged (5000× *g*, 15 min). Finally, the supernatant was poured into a dish in an oven at 110 °C overnight. The calculation was performed with the equations as follows.

Swelling power (%) = W wet sediment/(W sample − W residue) × 100(2)

Solubility (%) = W residue/W sample × 100(3)

W = weight (g)

### 2.10. Pasting Properties 

A rapid visco analyzer (RVA) (RVA4500, PerkinElmer Inc., Waltham, MA, USA) was used to determine the pasting properties of EFS according to the method by Panghal et al. [20]. The 3.5 g sample was weighed in the RVA canister and mixed homogeneously with 25 mL of distilled water. Heating was performed at a rate of 12 °C/min by heating at 50 °C for 1 min and 95 °C for 2.5 min. The rate of temperature reduction was similarly cooled from 95 °C to 50 °C at a rate of 12 °C/min with 3 min of holding at 50 °C. The peak (PV), breakdown (BV), setback (SV), trough (TV), final viscosities (FV), and pasting temperature (PT) were obtained by the instrument software.

### 2.11. X-ray Diffraction (XRD)

The sample was irradiated with Cu (D8, ADVANCE, Bruker Co., Billerica, MA, USA), and the diffractograms at angles 2θ set from 5° to 40° with a rate of 2°/min were collected at a target voltage of 40 kV and 30 mA. Such peaks were obtained with the XRD system software.

### 2.12. Total Phenolics Content (TPC) and Flavonoids Content (TFC)

Total phenolics were extracted, and total phenolics content was (TPC) determined according to the methods reported by Jogihalli et al. [21]. Gallic acid (GA) was used to draw the standard curve (y = 0.0057x + 0.0452, R^2^ = 0.9979) and TPC was expressed as mg of gallic acid equivalent per gram of CPs flour dry weight (mg GAE/g DW).

Total flavonoid content (TFC) was determined according to Jogihalli et al. [21]. Quercetin was utilized for making the standard curve (y = 0.0018x − 0.0013, R^2^ = 0.9989) and TFC was expressed as mg of rutin per one gram of CPs flour dry weight (mg RE/g DW).

### 2.13. Antioxidant Activity of EFS

The scavenging activity on the DPPH radical of the sample was measured with reference to the method of Huang et al. [22], with a slight modification. In brief, 100 μL of different samples and vitamin C concentrations were added with 100 μL of 0.2 mM DPPH, respectively. The blank was made by mixing 100 μL of different concentrations of samples with 100 μL of methanol (70%). It was mixed uniformly, and the reaction was carried out for 45 min at room temperature without light. Then, the absorbance at 517 nm was measured using a microplate reader (Agilent Technologies, Inc., Santa Clara, CA, USA). The DPPH radical scavenging rate was evaluated as follows

Scavenging rate (%) = {1 − (As − Ab)/Ac} × 100(4)

As is the absorbance of the sample,

Ab is the absorbance of the blank group,

Ac is the absorbance of the control group.

The reducing power measurements of the samples were measured according to the method of Huang et al. [22] with slight modifications. The different concentrations of samples and vitamin C were taken at 0.20 mL each and then mixed with 0.5 mL of phosphate buffer (0.2 M, pH 6.6) and 0.5 mL of potassium ferricyanide (1%, *w*/*v*). Next, the reaction was carried out in a water bath at 50 °C for 30 min. Then 0.5 mL of trichloroacetic acid (10%, *w*/*v*) was added and the mixture was centrifuged at 3000 rpm for 10 min. The above supernatant was taken at 0.5 mL, then added with 0.5 mL of deionized water and 0.1 mL of ferric chloride solution (0.1%, *w*/*v*), mixed, and reacted at room temperature for 10 min; the absorbance was measured at 700 nm.

### 2.14. Statistical Analysis

All experiments were performed in triplicates. All data were statistically significant with one-way ANOVA, and expressed as mean ± standard deviation (SD). The correlation coefficients by IBM SPSS Statistics (version 20, IBM SPSS Institute Inc., Armonk, NY, USA) were analyzed at a level of probability of *p* < 0.05. Origin software (Pro version 9.1, Origin Lab, Miami, FL, USA) was used to analyze the initial data and for plotting.

## 3. Results and Discussion

### 3.1. Comparative Analysis of Surface Morphology Structure of EFS

**Figure 1:**
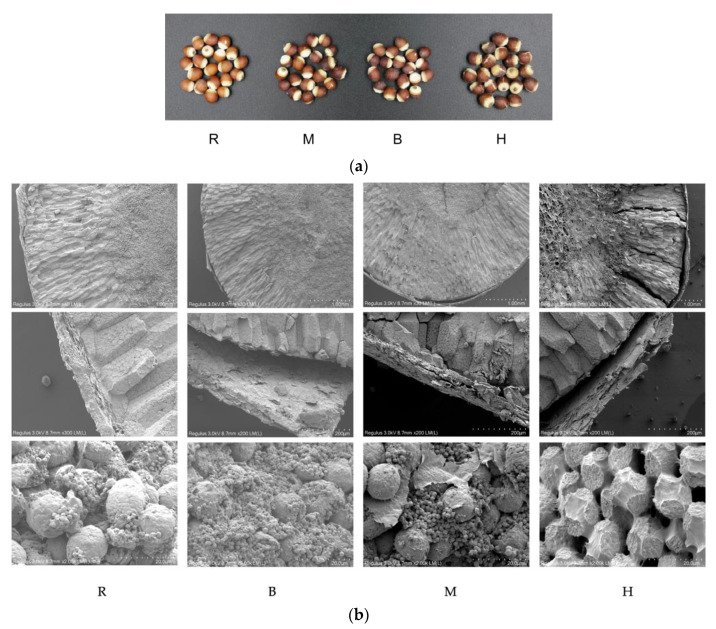
Surface morphology and microstructure of *Euryale ferox* seed (EFS). (**a**) Image of EFS after different thermal processing. (**b**) Scanning electron microscopy. R: Raw; M: Microwave; B: Baking; H: Industrial infrared heating-assisted fluidized bed (IHFH).

The appearance of raw EFS and treated EFS from three different thermal processing methods (baking, microwave, and IHFH) is shown in Figure 1a. Raw EFS appeared brown, and the seed hilum or scar appeared yellow. However, a significant color change was observed in the seed coat, and heat treatments followed the seed hilum as a darker brown color. Significantly, the surface of EFS showed different degrees of crack with heat treatments, which indicates that the present study was effective in destroying the surface structure of EFS by using three different heat treatment processes, which were similar to the previous studies [23,24]. It is shown that the roasted EFS of IHFH was prominent among the three thermal treatment methods.

### 3.2. Physical Properties of EFS Treated by Three Thermal Methods

The changes in the physical characteristics of raw and roasted EFS are presented in Table 1. All the thermally processed groups showed a significant decrease in bulk density and thousand-grain weight with a significant difference (*p* < 0.05) compared with raw EHS, in which the IHFH bulk density (618.4 g/L) and thousand-grain weight (264.56 g) showed the maximum decrease. In contrast, there was no significant difference between microwave (752.93 g/L and 270.24 g, respectively) and baking (754.27 g/L and 304.74 g, respectively). The probable reason might be the lower moisture content of roasted EHS compared with raw EFS. However, it was suggested from previous studies that kernel expansion of grains is due to disruption of the integrity of the macromolecular matrix (starch–starch, starch–protein, or proprotein–protein) either by heat or by the formation of porous structures in the endosperm structure [16,25]. It is worth mentioning that the phenomenon has been proved by observation of the microstructures (Figure 1b). Moreover, the puffing index (Table 1) showed a significant increase in value with IHFH processing and a significant difference compared with other groups (*p* < 0.05); meanwhile, it was also reflected in the appearance (Figure 1a) and microstructure (Figure 1b) of EFS. 

The kernel hardness is important because it affects energy requirements during milling [25]. The hardness of EFS decreased significantly (*p* < 0.05) with all three different thermal processes, with the most significant decrease (29.07%) in IHFH treatment. The possible reason was that IHFH provided a consistently suspended state, and enhanced EFS heated evenly, meanwhile promoting better kernel puffing, gelatinization of starch, generation of fissures, and disruption of the starch–protein structure in the cell wall and endosperm. Hence, the case of softening the texture of EFS is also found in the next section on the microstructures.

### 3.3. Microstructure Changes of EFS

Scanning electron microscopy (SEM) is widely applied to characterize the microstructure changes of cereal kernels in sub-micron resolution. The SEM micrographs of EFS cross-sections are presented in Figure 1b and the perisperm is a special structure of EFS and is important as the main nutrient source of seed germination and seedling growth [26]. Before heating and puffing, the raw material had an internal structure that was compact and relatively homogeneous, which exhibited a compact kernel structure, smooth cross-section, no gaps in the endosperm structure, and the pericarp is closed to the episperm. As expected, the heating and puffing treatment radically changed the microstructure of EFS. More gaps, fissures, and highly porous structures were found in the pericarp and exocarp intermediate and the endosperm by heat treatment, whereas IHFH was the most significant (Figure 1b). In addition, the pericarp was composed of thin fibrous tissue, which became looser after thermal treatments (Figure 1b). There were many starch complexes like “capsule” in the horny endosperm, which was encapsulated by proteins tightly (Figure 1(b2)). While in the silty endosperm, the starch complex was shown loosely, and more polygonal starch granules were coming out from the “capsule” after baking and microwave treatment (Figure 1b). However, there was no starch granule in the sample of IHFH treatment, where the starch and protein were bonded together and almost fused as a granule. Furthermore, lots of pores were produced between the granules (Figure 1b). It might lead to swelling, gelatinization, and breakdown of starch during IHFH, as well as denaturation of proteins.

### 3.4. Water Mobility and Distribution of EFS

**Figure 2:**
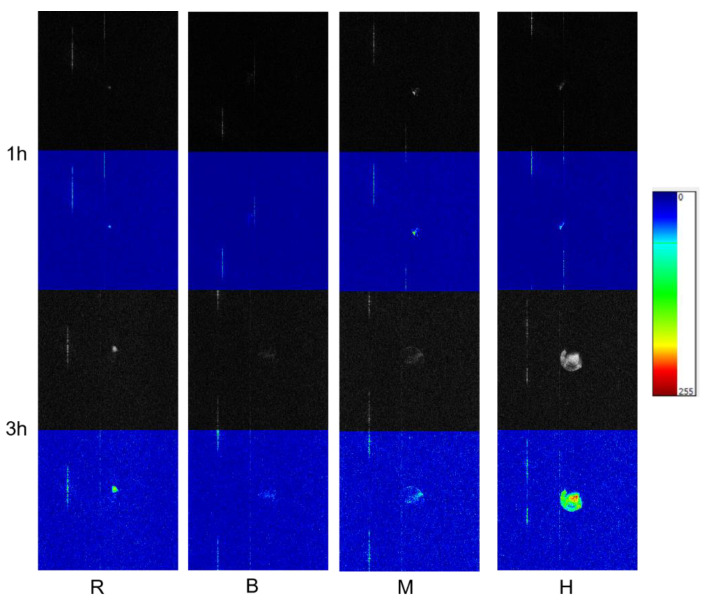
Low-field magnetic resonance imaging of EFS after different thermal processing. R: Raw; M: Microwave; B: Baking; H: Industrial infrared heating-assisted fluidized bed (IHFH).

Low-field magnetic resonance imaging (MRI) has been widely used as a noninvasive detection and analysis technique for water holding capacity, composition, internal water mobility and distribution, quality, and structural characterization during food processing [27]. It allows the experiment to be conducted without abolishing the shape or altering the physical properties of materials [28]. The MRI detected water mobility and distribution in cereal grains [29]. The T2-weighted images of raw and thermally processed EFS after soaking were presented in Figure 2. The color value ranging from 0 (black or blue) to 255 (white or red) represented that the signal intensity (proton density) of tissue water (hydrogen protons) varied from weak to strong. In other words, the greater the color value, the higher the moisture content. The white color area was small and not obvious on the image of raw EFS after soaking 1 h, which appeared in the middle of the EFS grain. After soaking for 3 h, the white color area increased insignificantly, which would be due to the difficulty of water mobility from outside to the inside of EFS. After three different thermal processing, the white color area became bigger, water mobility became easy after soaking, and the water content increased significantly, as shown in Figure 2. There were significant differences among the three thermal processing. The IHFH-treated EFS showed the most extensive white (red) areas with higher color values than the roasted and microwave-treated samples, while the red color appeared in the center of the EFS grains. It indicates that water quickly enters the interior from the middle of the grain with bright edges of the thermally processed grains, especially IHFH. It might be due to the fiber becoming loose in the seed coat and the protein denaturation, and the starch gelatinization in the horny endosperm expansion.

### 3.5. Color Characteristics of Roasted EFS Flour

The roasted EFS flour color change resulted, as shown in Table 1, in the raw group having L*, a*, and b* values of 88.18 ± 0.06, 0.79 ± 0.07, and 7.12 ± 0.22, respectively, which were significantly different compared with all thermally treated groups (*p* < 0.05). In this case, the raw group had the highest brightness (L*), which agrees with Figure 1a. The highest redness (a* as 3.35 ± 0.07) was found in the IHFH treatment, while microwave treatment had the highest yellowness (b* as 15.11 ± 0.34). The possible reasons might be attributed to the IHFH, which caused damage to the structure and appearance within the roasted EFS nuclei (as shown in Figure 1a, b). Meanwhile, the uniform heating behavior of IHFH promoted the Maillard reaction and browning reaction of EFS, while baking or microwaving was relatively weak. Furthermore, microwave increased the b* value, and a decrease in the b* value was observed via baking and IHFH treatment, which agrees with Bai et al. [16]. Some studies have reported color changes in grains and nuts during popping and roasting that also exhibited a decrease in brightness, which agrees with the results of this study [16,30,31]. According to Zameni et al. [32], the effect of different thermal treatments on basil seed color showed a decreasing trend for b* and an increasing trend for L* and a*. The color changes might be attributed to the nonenzymatic browning associated with degradation, oxidation, Maillard reaction, and caramelization of sugars during roasting and high-temperature processing of EFS [13]. Moreover, the total color difference ΔE was highest for the three different heat treatments with microwave treatment (9.21), followed by IHFH treatment (7.51), and the lowest value was baking (2.95) with significant differences (*p* < 0.05).

### 3.6. Water and Oil Absorption Capacity of Roasted EFS Flour

The water absorption capacity (WAC) of EFS (Table 1) increased significantly with the IHFH (2.77 g/g) treatment, followed by microwaved (2.55), while it did not increase significantly with the baking treatment (1.52); however, there was a significant difference in all groups (*p* < 0.05). In addition, the increase in WAC can be attributed to the gelatinization of the starch and the formation of porous structures during the heated roasting process, thus increasing the available capacity for adsorption. It was reported that microwave-roasted chickpeas showed an overall increase due to the high degree of starch damage caused by gelatinization and the seed porosity, which absorbed water via capillary action [13]. On the other hand, oven-roasting wheat grains revealed that the endosperm underwent destructive changes and empty spaces, which led to increased water absorption capacity, consistent with this study’s results [33]. 

Oil absorption capacity (OAC) results (Table 1) showed that microwave and IHFH treatments (0.93 g/g) were significantly different (*p* < 0.05) compared with Baking (0.89 g/g) and Raw (0.87 g/g), albeit with similar OAC values for each group. It has been reported that changes in OAC during processing were associated with the solubilization and dissociation of proteins into subunits at the same time as the increase or decrease in polar and nonpolar binding sites, which contribute to the OAC increase [31]. In addition, this study found that roasted EFS flour OAC increased with microwave and IHFH treatments, which might be attributed to the increased porous structure and physical entrapment of the oil on the non-polar side chains of the protein [34].

### 3.7. Swelling Power and Solubility of Roasted EFS Flour

Swelling power (SP) reflects the magnitude of the interaction between the starch chains within the crystalline and amorphous regions during the heating process [35]. This study’s SP of roasted EFS flour decreased significantly (Table 1). The lowest SP (7.02%) was found for IHFH processed, while the others were microwave, baking, and raw groups in descending order, which showed significant differences (*p* < 0.05). However, the decrease in SP of roasted EFS flour may be related to the pasting temperatures; there were reports of differences in pasting temperatures leading to accelerated molecular rearrangements or degradation of amylopectin molecules [36,37]. In terms of solubility, the roasted EFS flour showed increased solubility. In particular, the microwave-treated roasted EFS flour showed the highest solubility of 6.10%, followed by IHFH (5.72%), which showed a significant difference (*p* < 0.05) compared with the Baking (3.35%) and Raw (2.82%) groups. In addition, it was proposed that in a limited moisture environment at high temperatures, starch and protein would compete for water uptake; yet, the heat treatment denatured the protein, which disrupted the hydrogen bonds and caused the starch molecules to bond together, which led to partial gelatinization [38]. The gelatinization starch formed a barrier as it surrounded the intact ungelatinized starch granules, which retarded water absorption and decreased the starch’s solubility [37,39].

### 3.8. Pasting Properties

Regarding the pasting properties of the different heat-treated roasted EFS flours (Table 1), the pasting temperature increased from 82.74 °C to 83.49–84.82 °C with IHFH treatment being the highest. Microwave treatment was the second, followed by the baking, and significant differences were observed in each group (*p* < 0.05). It was indicated by the increased pasting temperature that the bonding within the heat-treated EFS was strengthened [17]. Simultaneously, the pasting properties of the raw EFS were destroyed by heat treatment. It has been reported that the peak viscosity represents the water retention capacity of starch granules, as reflected in the resistance of swollen starch granules to shear forces and the swelling properties of starch granules [40]. Notably, it was reported that the heating rate of microwave cooking is very high, thus limiting the water absorption and expansion of starch granules; simultaneously, the vibrational motion of water molecules and the reorientation of other polar molecules to the oscillating microwave field both affect the pasting efficiency of starch in microwave cooking [38].

**Figure 3:**
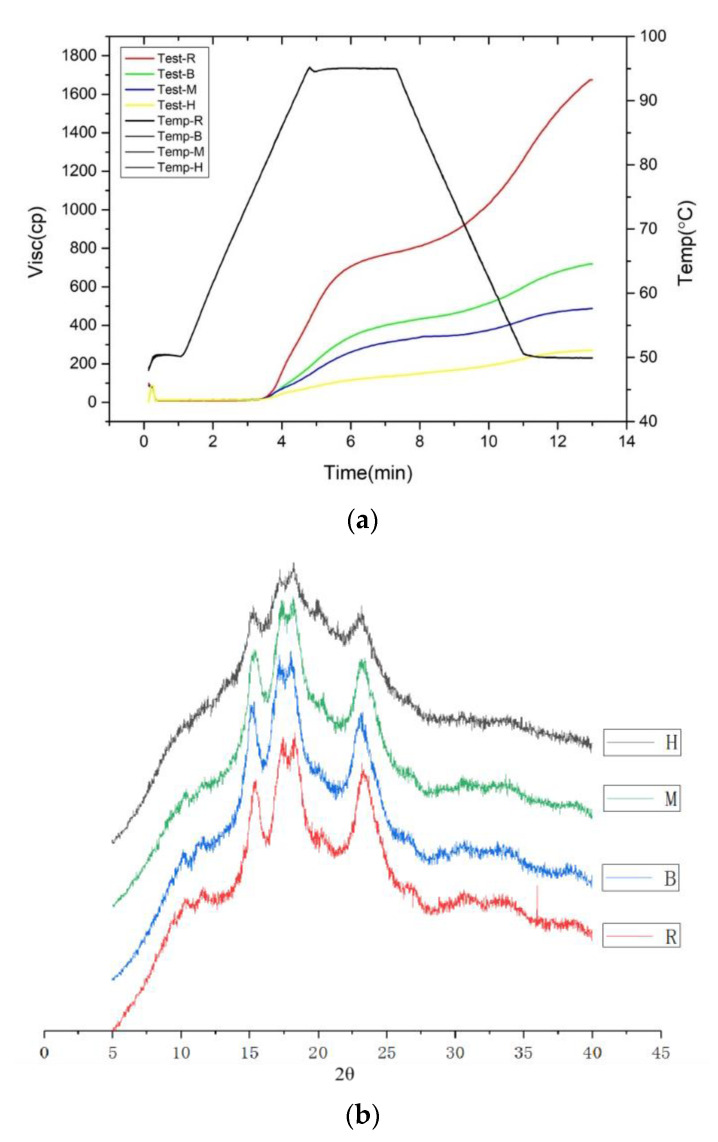
Physical-functional properties of roasted *Euryale ferox* seed (EFS) flour. (**a**) Viscosity curve of the raw, (**b**) X-ray diffraction pattern, and thermal processing EFS flours. R: Raw; M: Microwave; B: Baking; H: Industrial infrared heating-assisted fluidized bed (IHFH).

In the peak viscosity (PV), compared with the raw group, IHFH decreased the most, followed by microwave and then baking, which showed a significant difference in the range of decrease from 763 cp to 139 cp (*p* < 0.05). The trend of final viscosity (FV) was similar above, from a high minus small amount to low in the order of IHFH, microwave, and baking. However, compared with the raw group, the decrease range was from 1854 to 268.67 cp, with a significant difference (*p* < 0.05). In addition, the FV of roasted EFS flour decreased, which means a lower tendency of retrogradation in reverse. Unfortunately, neither trough viscosity (TV), break down viscosity (BD), nor set back viscosity (SV) were detected in this study (Table 1 and Figure 3a). The previous study reported that the possible reasons for the decrease in the viscosity profile were attributed to hard unfractured swollen granules, granule size, and a decrease in the percentage of amylose leached with heat treatment severity [36]. The difference in the viscosity was attributed to the different degrees of damage after the three thermal treatments. Investigations have shown that wheat products such as cakes and noodles produced from milled heat-treated kernels will cause higher viscosity, thus improving mouthfeel and texture [41,42].

### 3.9. Crystal Form and Relative Crystallinity

Evidence of X-ray diffractograms (XRD) is a useful method to assess and quantify long-range crystallization order in starch [43]. The detected results of XDR (Figure 3b) showed a typical A-type crystalline polymorph for all groups, which had characteristic peaks with strong intensities at 15° and 23°, followed by an unresolved doublet peak near 17° and 18°. It proves that EFS has the same crystalline polymorph as many kinds of cereal such as corn and rice [44] and has the same result as previous studies about EFS [17]. Hence, the baking and microwave groups showed no alteration in the overall X-ray diffraction pattern (compared with the raw group) caused by the heat treatment, in contrast to the IHFH treatment, which showed a significant decrease in the intensity of the characteristic peaks compared with the other three groups.

### 3.10. Total Phenolics Content (TPC) and Total Flavonoids Content (TFC) of EFS

**Figure 4:**
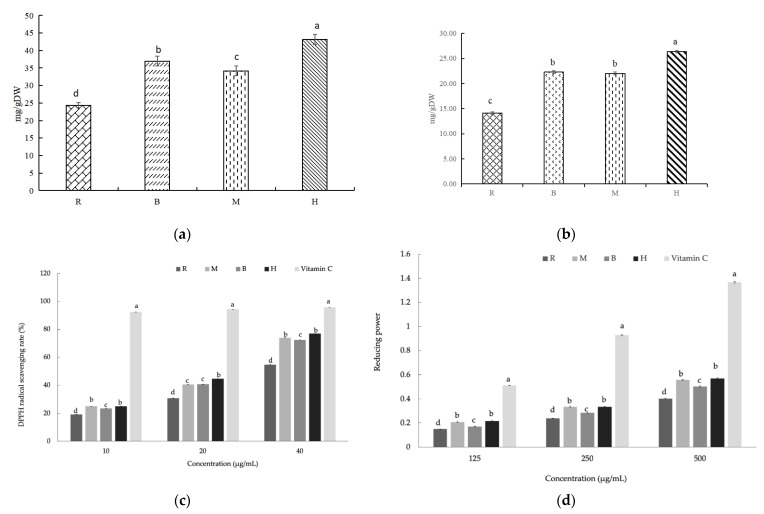
Functional components content and antioxidant activity of roasted *Euryale ferox* seed (EFS) flour. (**a**) Total phenolics content (TPC), (**b**) flavonoid content (TFC), (**c**) reducing power, and (**d**) DPPH free radical scavenging rate (%). R: Raw; M: Microwave; B: Baking; H: Industrial infrared heating-assisted fluidized bed (IHFH). Different lowercase letters represent significant differences (*p* < 0.05).

Phenolic compounds and flavonoids are widely regarded as the main bioactive components in EFS, which display remarkable health-promoting effects on antioxidants and antineoplastics [31]. In this study, the TPC results showed (Figure 4a) that there were significant differences (*p* < 0.05) with the highest increase in IHFH treatment (86.63%) followed by baking (57.71%) and then microwave (55.87%) compared with the raw group. Notably, it was reported that the phenolic content of roasted chickpea samples significantly increased compared with unroasted ones; it was hypothesized as a possible result of heat induced and the formation of extractable phenolic compounds, which are similar to the results of this study [13]. The other possible reason might be that dried heat disrupts the outer layer of the grain and the endosperm structure, which facilitates the release of bound phenols from the cross-linked network by disrupting the cell structure [16].

The TFC also showed a similar trend as described above, with significant differences (*p* < 0.05) in all thermal treatments compared with the raw group (Figure 4b). In particular, a maximum increase was observed for IHFH treatment (76.90%), followed by baking (51.74%) and then microwave (40.34%). Well-known flavonoids are heat-sensitive phenolic compounds; thus, heat exposure to the roasting process may cause a decrease in TFC. Yet, it has been reported that a significant reduction in TFC was observed with low microwave power, while a slight increase in TFC was observed with increasing time at high power levels [13]. It has been previously reported that the increase in TFC of dry heat-treated grains may be due to the partial disruption of the cell structure, resulting in the release of compounds [45]; subsequently, these compounds were diffused from the seed coat to the kernel.

### 3.11. Antioxidant Activity of EFS

The antioxidant activities of EFS extracts were evaluated by DPPH free radical scavenging activity and reducing power (Figure 4c,d). The present study found that DPPH radical scavenging activity increased significantly with EFS concentrations from 10 to 40 µg/mL and was increased considerably with thermal treatment (Figure 4c). The performance of IHFH (from 25.00% to 76.87%) and microwave (24.99% to 73.86%) were better, followed by baking (25.56% to 72.40%), yet there was a significant difference compared with the control group (*p* < 0.05). The reducing power showed a dose-dependent relationship with EFS concentrations (125 to 500 µg/mL) (Figure 4d), ranked from high to low as IHFH (from 0.22 to 0.57) and microwave (0.21 to 0.56) were better, followed by baking (0.17 to 0.50). EFS showed an increasing trend in reducing power in all cases by thermal treatments, with a significant difference compared with the control group (*p* < 0.05). The proposed reasons might be attributed to EHS seed coats being rich in TFC, TPC, and active antioxidant compounds [2]. At the same time, thermal treatments proceeded to damage the cell walls of the seed coats, which promoted the diffusion of the compounds from the coats to the kernels. Moreover, it has been suggested that the dry thermal treatment disrupts the cell structure of whole grain highland barley to promote antioxidant release [16]. Overall, during the dry heat treatment, it causes the Mailard reaction and caramelization, enhancing antioxidant activity and providing color, aroma, and flavor [38]. Hence, the dual benefits of improved sensory characteristics and enhanced bioactive contents were observed.

## 4. Conclusions

The present research demonstrated that the thermal treatment influenced the physicochemical and nutritional properties of EFS. The bulk density, thousand kernel weight, and hardness were decreased significantly, and the microstructure was shown differently. There were more gaps, fissures, and highly porous structures between pericarp and episperm and in the endosperm after thermal treatments. The color changed significantly. Both WAC and OAC of EFS increased significantly. The SP of the EFS decreased significantly, while the solubility of roasted EFS flour increased significantly after thermal processing. The pasting properties of raw EFS were disrupted after three thermal treatments. The overall X-ray diffraction patterns were not affected significantly after thermal treatments. TPC and TFC increased significantly (*p* < 0.05) after thermal processing three times. THE DPPH free radical scavenging activity and reduction of the power of EFS increased significantly (*p* < 0.05) after thermal processing three times. Overall, both microwave and IHFH have the best performance as heating methods; however, the microwave has the most potential for application in the family for general people without a professional background. This study provides valuable insights and promotes EFS to develop healthier foods in the marketplace. Moreover, a theoretical basis is provided for future research and the application of EFS.

## Figures and Tables

**Table 1:** Effect of thermal treatments on the physical, color, physical-functional, and pasting properties of roasted *Euryale ferox* seed (EFS) flour.

	Raw	Baking	Microwave	Industrial Infrared Heating-Assisted Fluidized Bed (IHFH)
Physical properties				
Bulk density (g/L)	761.93 ± 1.15 a	754.27 ± 0.90 b	752.93 ± 1.47 b	618.43 ± 1.30 c
Puffing index	1.00 ± 0.00 b	1.01 ± 0.025 b	1.01 ± 0.021 b	1.23 ± 0.065 a
Thousand weights (g)	309.48 ± 1.05 a	304.74 ± 0.95 b	270.24 ± 1.88 c	264.56 ± 1.45 d
Hardness (N)	57.90 ± 2.53 a	52.95 ± 1.59 b	49.07 ± 3.95 c	46.44 ± 1.83 c
Color properties				
L*	88.18 ± 0.80 a	86.02 ± 0.40 b	83.75 ± 0.33 c	81.70 ± 1.28 d
a*	0.79 ± 0.09 d	2.10 ± 0.16 b	1.71 ± 0.02 c	3.35 ± 0.02 a
b*	7.12 ± 0.14 d	8.53 ± 0.50 c	15.11 ± 0.20 a	9.91 ± 0.19 b
ΔE	-	2.95 ± 0.26 c	9.21 ± 0.13 a	7.51 ± 0.88 b
Physical-functional properties				
Water absorption capacity (g/g)	1.43 ± 0.05 c	1.52 ± 0.07 c	2.55 ± 0.05 b	2.77 ± 0.06 a
Oil absorption capacity (g/g)	0.87 ± 0.01 b	0.89 ± 0.01 b	0.93 ± 0.01 a	0.93 ± 0.02 a
Swelling power (%)	9.16 ± 0.06 a	8.05 ± 0.08 b	7.44 ± 0.12 c	7.02 ± 0.08 d
Solubility (%)	2.82 ± 0.04 d	3.35 ± 0.05 c	6.10 ± 0.08 a	5.72 ± 0.07 b
Pasting properties				
PV (cp)	763.00 ± 4.58 a	403.33 ± 3.06 b	309.00 ± 1.00 c	139.00 ± 3.00 d
TV (cp)	ND	ND	ND	ND
BD (cp)	ND	ND	ND	ND
FV (cp)	1854.00 ± 13.75 a	725.33 ± 6.03 b	486.33 ± 3.06 c	268.67 ± 1.53 d
SB (cp)	ND	ND	ND	ND
PaT (°C)	82.74 ± 0.06 c	83.49 ± 0.04 b	83.84 ± 0.03 b	84.82 ± 0.20 a

* Means are expressed as means ± standard deviation (SD). The different letters in each analysis represent significant differences (*p* < 0.05). Each sample was triple analyzed. ND means non-detected.

## Data Availability

Not applicable.
